# Drop-on-Demand Single Cell Isolation and Total RNA Analysis

**DOI:** 10.1371/journal.pone.0017455

**Published:** 2011-03-11

**Authors:** Sangjun Moon, Yun-Gon Kim, Lingsheng Dong, Michael Lombardi, Edward Haeggstrom, Roderick V. Jensen, Li-Li Hsiao, Utkan Demirci

**Affiliations:** 1 Demirci Bio-Acoustic-MEMS in Medicine (BAMM) Laboratory, Center for Bioengineering, Brigham and Women's Hospital, Harvard Medical School, Boston, Massachusetts, United States of America; 2 Renal Division, Brigham and Women's Hospital, Harvard Medical School, Boston, Massachusetts, United States of America; 3 Electronics Research Laboratory, Department of Physics, University of Helsinki, Helsinki, Finland; 4 Department of Biological Sciences, Virginia Tech, Blacksburg, Virginia, United States of America; 5 Harvard-MIT Division of Health Sciences and Technology, Massachusetts Institute of Technology, Cambridge, Massachusetts, United States of America; University of Sao Paulo - USP, Brazil

## Abstract

Technologies that rapidly isolate viable single cells from heterogeneous solutions have significantly contributed to the field of medical genomics. Challenges remain both to enable efficient extraction, isolation and patterning of single cells from heterogeneous solutions as well as to keep them alive during the process due to a limited degree of control over single cell manipulation. Here, we present a microdroplet based method to isolate and pattern single cells from heterogeneous cell suspensions (10% target cell mixture), preserve viability of the extracted cells (97.0±0.8%), and obtain genomic information from isolated cells compared to the non-patterned controls. The cell encapsulation process is both experimentally and theoretically analyzed. Using the isolated cells, we identified 11 stem cell markers among 1000 genes and compare to the controls. This automated platform enabling high-throughput cell manipulation for subsequent genomic analysis employs fewer handling steps compared to existing methods.

## Introduction

For stem cell characterization, understanding single cell level functional genomics has become increasingly important [1], [2], [3], [4], [5], [6], [7], [8]. As new regenerative therapies using tissue engineering [2] emerge, the existence of tissue-specific stem cells in adult organs has extensively been investigated in bone marrow, skin, heart, muscle, pancreas, lungs, and the nervous system. However, the characterization of differentiated progeny has been hampered by the lack of cell markers and low viability of the purified cells [9]. For instance, single cell transplantation methods can significantly benefit from efficient cell isolation and handling techniques [10]. Recent advances in mRNA amplification and cell sorting technologies offer insights into single cell genomics [1], [2], [3], [4], [5], [6], [11]. However, single cell level genomic studies require amplification by a factor of a billion to reach detection levels from a few femtograms of mRNA present in a single cell. Therefore, to accurately profile single cell genes from a heterogeneous cell solutions or a tissue sample, it is essential to minimize RNA contamination from surrounding cells by enriching the fraction of the target cell type.

**Figure 1:**
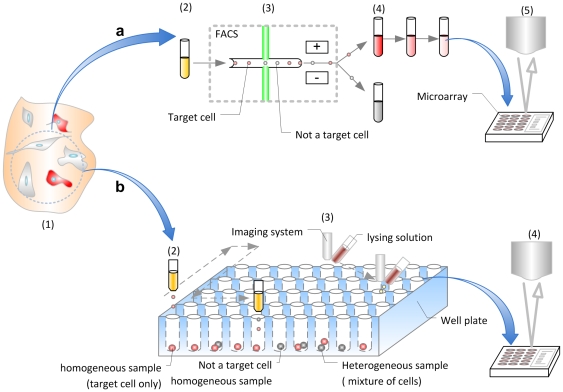
Description of a conventional procedure for total RNA expression analysis versus the technique described. (**a**) Conventional single cell isolation method for total RNA expression analysis. 1) Heterogeneous sample was collected from a specific tissue, macro-dissection, 2) cells were stained with a specific antibody, 3) target cells were collected with conventional FACS, 4) multiple dilution steps generated a small population of target cells, 5) total RNA gene expression analysis with microarray. (**b**) Drop-on-demand total RNA analysis approach. 1) Heterogeneous sample was collected, 2) cells were stained with antibody and patterned with cell-encapsulating droplets, 3) specific homogeneous samples containing target cells were produced by a cell droplet patterning platform; homogeneous samples were identified by an automated imaging system, 4) total RNA gene expression analysis with microarray.

Cell pattering and encapsulation in droplets is a challenging and exciting field with multiple possible applications including tissue printing [12], [13], cell sorting [14], and cryobiology [15]. Several approaches have been developed to isolate single cells (**Table 1**). The most common methods are microscale cell manipulation [16], serial dilutions of a culture or a co-culture of cells [17], laser capture microdissection (LCM) [18], and fluorescence-activated cell sorting (FACS) [19]. These methods have challenges with complexity, time consumption and inefficiencies in isolating cells that are contamination-free and viable. In addition, histological methods can damage mRNA both in frozen and paraffin-embedded sections [20]. Traditional FACS and LCM require large sample volumes (milliliters), and utilize expensive instruments used by skilled operators (**Fig. 1a**). FACS can sort cells, at a single cell level in nanoliter volumes. However, FACS does not pattern these cells. Recently, these traditional technologies have been modified and adapted towards microfluidics [21], [22], [23], [24], [25], [26], [27]. Microengraving [27], [28] has low complexity; it rapidly loads individual cells, and creates low mechanical stress during cell loading. However, it suffers from limited control over number of cells per well due to manual cell loading process. These new technologies have potential for single cell genomic analysis of target cells, *e.g.* stem cells and uncultured organisms [11]. However, these capabilities arrive at a substantial cost in increased design complexity, development cost [29]. Further, there are challenges to control the number of cells deposited to a predetermined location. The microfluidic systems are also great tools to handle and sort cells. Although the cell handling processes have been simplified in microfluidic systems, cell tracking for sorting on chip still requires peripheral setups followed by subsequent cell separation steps. As the heterogeneity of sample increases, the types of cells that need to be tracked in the sample also increase. The outcome is that the tracking system requires more complex peripheral setups and it can require high-end computerized controls which has an impact on scalability. A great example of such systems is best demonstrated by Quake and Hong [29], [30], [31], [32]. To address these challenges, we developed a simple, high-throughput platform for single cell isolation with direct access to patterned cells (**Fig. 1b**). The methodology is based on a “drop-on-demand” cell patterning technology that follows simple random sampling (SRS) [33], [34], [35]. The system patterned an array of 10×10 droplets that encapsulated single cells from a heterogeneous cell mixture at a high-throughput of within 4 seconds. Subsequently, imaging systems with a wide field of view are available to monitor the patterned droplet array within a few seconds showing positions of target cells [36]. Since the droplets are printed onto a glass surface, each encapsulated cell of interest can then be accessed freely.

**Table 1:** Performance comparison with conventional methods: isolating single cell's from a heterogeneous cell mixture.

	Categories	FACS [39]	Microfluidics (μFACS [22], [23], [24], [25], [26], [40])	Single cell droplet
Cell viability*	Overall process viability	80%	97%	97%
Platform	Cell isolation method	Electrostatic deflection	Fluidics	Droplet array
Throughput	Parallel processing	Low	Medium	High
	Unit processing time	∼1 msec/cell	∼3 sec/cell	∼50 µsec/cell
Usage	Heterogeneous sample	Yes	Yes [39]	Yes
	Single cell accessibility	Tube/dilution	Closed channel/chamber	Open-top nanoliter droplet array
	Application	Cell sorting	Single cell PCR	RNA analysis

*Cell viability = post processing cell viability/reservoir cell viability.

**Figure 2:**
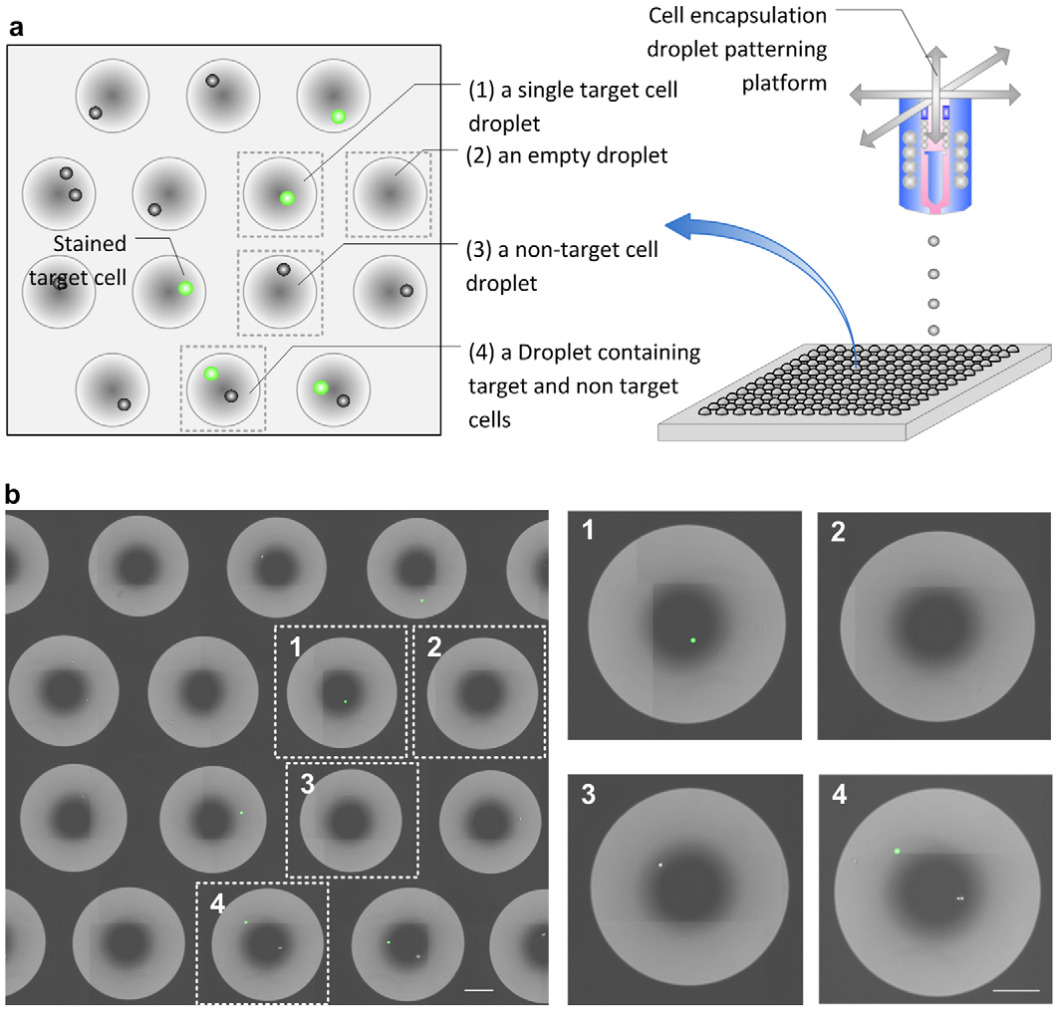
Patterned droplets. (**a**) Schematic droplet subarray showing four different cases of patterned droplets. Green colored circles indicated target cells. (**b**) Portion of a droplet array showing examples of each of the four different cases of patterned droplets, 1) single target cell droplet, 2) an empty droplet without cells, 3) a non-target cell droplet, and 4) a heterogeneous droplet containing target and non-target cells. Green fluorescent stained cells were target cells. Scale bars, 200 µm.

In this paper, we experimentally and theoretically analyzed the cell encapsulation process in microdroplets. We analyzed the cell encapsulating droplets for cell viability and performed genomic analysis on the printed stem cells and compared them to the non-patterned controls. Here, we present the first time genomic analysis performed on cells patterned using cell printing indicating that the cells are genomically functional through the printing process compared to the controls. We used immunostaining prior to patterning to distinguish target cells within the heterogeneous population. Based on the optical images, each patterned droplet fell into one of the four categories: a droplet containing (i) a single target cell, (ii) no cells (empty droplet), (iii) a non-target cell, or (iv) multiple cells (of either the target and non-target cell type) (**Fig. 2b**). Then, we performed RNA extraction from cells within nanoliter droplets for genomic analysis. This permits high-throughput processing and reduction in time required for isolation of cells. Such novel methods can enable rapid biomarker studies in systems biology research [37].

## Results and Discussion

We evaluated our drop-on-demand single cell patterning platform by measuring droplet volume, number of cells encapsulated per droplet for different cell loading concentrations and target cell content. Droplet volume was measured by two independent techniques: stroboscopic imaging in air and wetting contact angle measurements on a glass surface. Stable droplet generation at 25 Hz was achieved by employing a 34.4 kPa ejection pressure at 1.25 mPa·s viscosity. Under these conditions, the droplet diameter in air depended on the valve opening duration. The average droplet diameters in air were 220±9 µm, 245±12 µm, and 285±18 µm, corresponding to valve opening durations of 50 µs, 55 µs, and 60 µs, respectively. A 55 µs valve opening duration was chosen as the minimum value required to avoid clogging and formation of satellite droplets. We printed 50×50 array of droplets at a rate of 25 droplets per second. The array density was one droplet per mm^2^ with a 525±15 µm droplet spread diameter on the surface. This droplet spread diameter depended on the surface tension (28.3° contact angle on a glass slide) and droplet volume (7.6±0.6 nanoliter per droplet; see **Materials and Methods**, *Drop-on-demand single cell patterning*).

**Figure 3:**
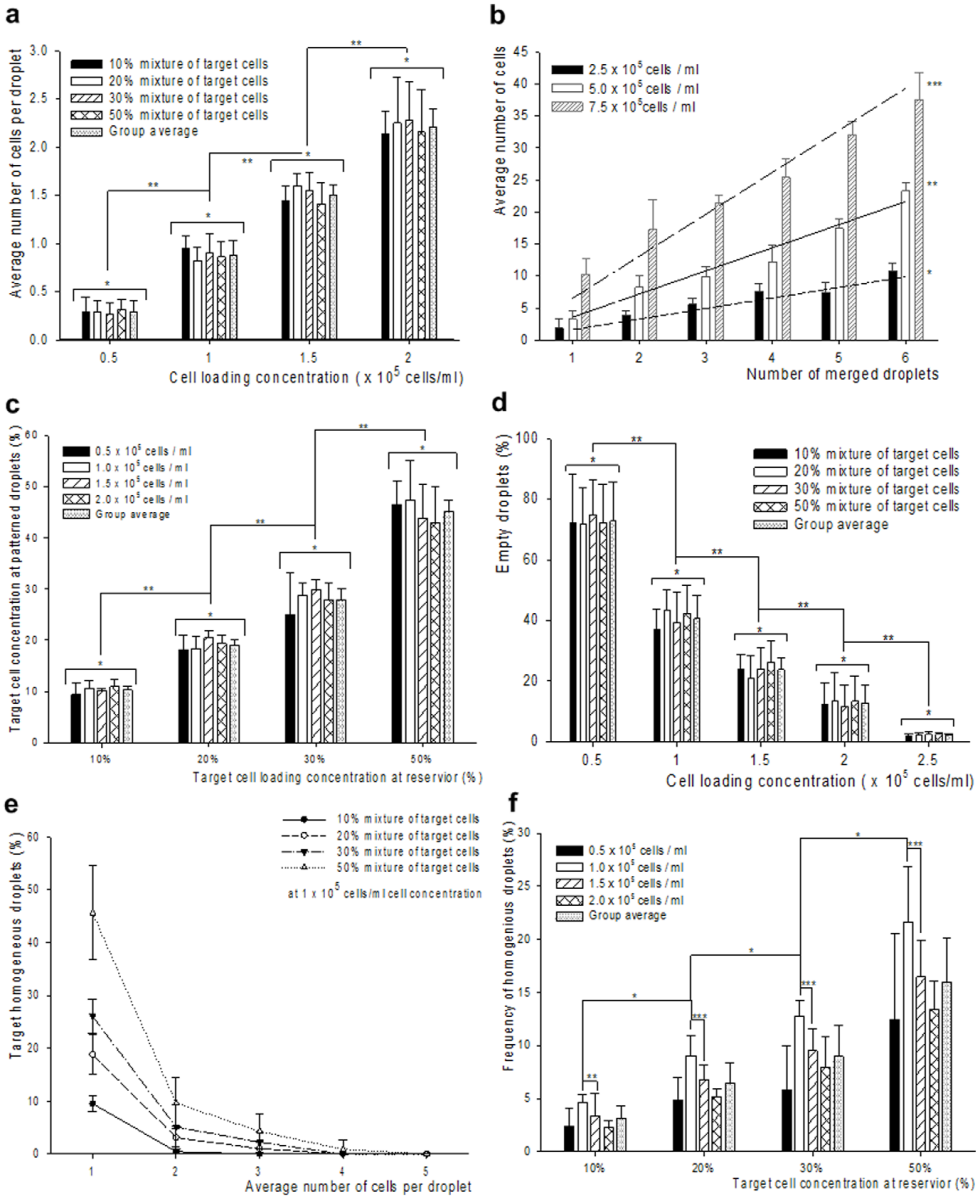
Homogeneous droplet analyses. (**a**) Droplets are generated at cell concentrations: 0.5×10^5^, 1.0×10^5^, 1.5×10^5^, and 2.0×10^5^ cells/ml and they contained 0.28±0.12, 0.88±0.14, 1.50±0.10, and 2.20±0.19 cells per droplet, respectively (mean ± standard deviation). The droplets were ejected using two controlled parameters: 34 kPa nitrogen gas pressure and 55 µs valve opening period. Values were obtained from 5 groups, each containing 100 droplets. Each group was tested by single factor analysis of variance (ANOVA). F(3,16) = 0.15, 0.58, 1.22, and 0.16, n = 5 mean values per each group, *p>0.33. (Student's t-test between target cell concentrations: **p<0.0004, n = 5 mean values of 4 concentrations). (**b**) Number of cells in droplets was obtained at cell concentrations: 2.5×10^5^, 5.0×10^5^, and 7.5×10^5^ cells/ml. Regression coefficients, R^2^ values, were *0.93 (1.7 cells/drop), **0.96 (3.6 cells/drop), and ***0.92 (6.6 cells/drop), n = 28 droplets. (**c**) Comparing target cell concentration in the reservoir and in the droplet pattern. Average and standard deviation of 10% to 50% target cell concentration in the reservoir were 10.3±0.6, 19.0±1.0, 27.8±2.0, and 45.1±2.1. F(3,16) = 0.82, 1.38, 0.98, and 0.52, *p>0.29, ANOVA for each cell concentration (student's t-test for each target cell concentration: **p<0.001, n = 5 mean values of 4 different target cell concentrations). (**d**) Fraction of empty droplets in droplet array as a function of cell concentration. Average and standard deviation of empty droplets for 0.5 to 2.5×10^5^ cells/ml concentrations were 72.8±12.8%, 40.6±7.7%, 23.8±4.0%, 12.7±5.8%, and 2.2±0.3%, respectively. F(3,16) = 0.05, 0.64, 0.56, 0.05, and 0.67, *p>0.58, ANOVA for each target cell concentration (student's t-test for each cell concentration: **p<0.016, n = 5 mean values of 4 different target cell concentrations). (**e**) Fraction of target homogeneous droplets among the patterned droplets was calculated as the ratio between the number of cells in homogenous droplets and the overall number of droplets for different cases: 1, 2, 3, 4, and 5 cells per droplet. This excluded the empty droplets. At 1.0×10^5^, for the case of one cell per droplet, the average and standard deviation values of homogeneity were 9.5±1.4%, 18.8±3.7%, 26.0±3.1%, and 45.5±8.8% for 10 to 50% target cell concentration. The fractions were 0.4±0.9%, 3.1±2.1%, 5.1±4.2%, and 9.6±4.8% for droplets containing two cells. (**f**) Frequency of homogeneous droplets based on cell concentration. This analysis includes the empty droplets. Among the different target cell concentrations, the 1×10^5^ cells/ml concentration showed 4.7±0.6%, 9.0±1.9%, 12.7±1.6%, and 21.6±5.2% homogeneous droplet occurrences. F(1,8) = 22.7, 11.2, and 13.3, *p<0.01, ANOVA for 1×10^5^ cells/ml through 10 to 50% target cell concentration (student t-test: **p = 0.12, ***p<0.05, n = 5). Error bars represented the standard deviation of the mean. The p values in student's t-test were calculated based on two-sided distributions with unequal variances.

Using these droplet generation parameters, we investigated the effect of the cell loading concentration on the patterned droplets. We obtained both experimental values and developed statistical models that describe the cell encapsulation process. Heterogeneous mixtures of mouse embryonic stem cells (mESCs) were prepared consisting of 10% to 50% stained target stem cells. The average number of target cells per droplet was calculated over 100 droplets (see **Materials and Methods**, *Cell preparation and staining*). We tested cell loading concentrations ranging from a 0.5×10^5^ to 7.5×10^5^ cells/ml. Our theoretical and experimental results indicated that the 1.0×10^5^ cells/ml concentration was optimal to encapsulate single cells in droplets, since the resulting average number of cells per droplet and the standard deviation were 0.88±0.14 (**Fig. 3a**). The model (see **Fig. S5a** online) agrees with the experimental results **Fig. 3a**. These both indicate that the maximum single cell encapsulation event is likely to take place at the 1×10^5^ concentration. These statistics correspond to the case of single cell encapsulation within a droplet (with 36.8% probability), followed by other cases of an empty droplet (**Fig. 3e**, 36.8%), and a droplet containing two cells (**Fig. 3e**, 18.4%). Further, the number of cells per droplet was found to be independent of the target cell fraction (**Fig. 3a**, 10–50%) in the heterogeneous cell mixture. We observed that the standard deviation for the number of encapsulated cells per droplet was higher than ±1.5 cells when the cell loading concentrations exceeded 2.5×10^5^ cells/ml (**Fig. 3b**). However, the number of cells per droplet obtained by merging multiple droplets by ejection to the same location showed smaller standard deviation compared to ejecting a single droplet at a higher cell loading concentration (less than ±0.7 cells/droplet, **Fig. 3b**). This result is due to a small cell-to-droplet volume fraction of 1.7% using 7.6 nanoliter droplets. As we increase number of droplets, the sampling process fulfills the law of large numbers (LLN, n = 106, 90% confidence level and 15% tolerance), the cell encapsulation mechanism can be treated as a random cell sampling process from a large population of cells, *i.e.* Student's t-test and single factor analysis of variance (ANOVA). These statistical results as shown in **Fig. 3** indicate that our random cell encapsulation and patterning methods follow the central limit theorem (CLT) [33], [34], [35]. This allows us to conclude that the average and standard deviation of sampled droplets can be used for statistical estimation of entire sample volume. This approach may provide a powerful tool for sampling single target cells in a high-throughput manner without searching the entire cell population in a reservoir.

We investigated the viability of the cells patterned in droplets. The reservoir cell viability was 96.7±0.5% using a 10 µl volume at 7.5×10^5^ cells/ml. Following the 1.7 minutes of patterning process using a 19 µl sample, cell viability was observed to be 93.8±1.1% in a 50×50 array of cell encapsulating droplets. When the optimal cell concentration for single cell patterning (1.0×10^5^ cells/ml) was used, the patterned cell viability was 97.0±0.8%. The relatively higher patterning viability is due to reduced droplet packing density, which minimizes possible exposure to mechanical shear forces at the valve during cell encapsulation. Underlying this high viability was the low (1.7%) cell-to-droplet volume fraction we used during encapsulation process. Furthermore, we confirmed that patterned cell viability does not depend on the target cell concentration in the reservoir (see **Fig. S4** online).

Based on our statistical analysis (see **Materials and Methods** *Statistical Modeling*), the single target cell encapsulation process followed Poisson distribution and matched with 90% confidence level and 15% tolerance. These results show that our system can be used to encapsulate single target cells from a heterogeneous solution that has 9±1% target cells by using only 10×10 array of droplets. This reduces time to reach to target cells without searching the entire sample volume in the ejection reservoir.

We validated that our patterning method produces droplet arrays that conform to CLT by comparing the fraction of target cells in the reservoir to the fraction in the patterned droplet array. The comparison is conducted using 10×10 droplet array subset randomly chosen from a 50×50 patterned droplet array. The fraction of target cells in the reservoir cell mixture (10%) was essentially mimicked (10±2.2%) in the patterned droplet subarrays (**Fig. 3c**). The other heterogeneous samples (20%, 30%, and 50%) agreed with this result. Therefore, evaluating a 10×10 droplet array allowed us to infer the target cell concentration in the reservoir. The fraction of empty droplets was also investigated with concentrations ranging from 1.0×10^5^ to 2.5×10^5^ cells/ml. Percentage of empty droplets determines the expected yield when searching for target cells (*i.e.*, cells to be isolated for further mRNA extraction and analysis, **Fig. 3d**). At a low cell concentration, 0.5×10^5^ cells/ml, the probability of patterning empty droplets was highest (72.8±12.8%). We choose 1.0×10^5^ cells/ml concentration to pattern single cell encapsulating droplets. The fraction of empty droplets at this concentration ranged from 32.9% to 48.3% over the range of cell mixture compositions studied. Despite these values, high-throughput target cell isolation was achieved with the automated rapid patterning capability.

The patterning process samples 10×10 of droplets (of total volume 0.76 µL) out of the total reservoir volume of 100 µL. While characterizing the statistics governing the patterned target cell droplets printed from a heterogeneous cell mixture, we observed agreement (0.9%∼7.0% difference) between the reservoir mixture composition and the composition observed in patterned subarrays for 10% to 50% target cell containing mixtures (**Fig. 3e**). Starting from a cell reservoir containing a 10% fraction of target cells, a target homogeneous droplet fraction of 9.5±1.4% was observed in a 10×10 droplet subarray at a reservoir cell density of 1.0×10^5^ cells/ml. Experimental results indicate that isolation of “a droplet containing a single target cell” within a 10×10 array is likely (at >90% confidence level) for target cell fractions down to 1% (**Fig. 3e**). This lower limit of sampling fraction, 1% v/v, assumes the empty droplet percentage to be at the maximum value that we experimentally observed to be as 48.3% at 1.0×10^5^ cells/ml concentration. Moreover, when a droplet contained more than three cells, the fraction of droplets containing only target cells became zero in the 10×10 droplet subarray. We also experimentally observed the highest probability to pattern homogeneous droplets at the reservoir concentration of 1.0×10^5^ cells/ml. At the 1.0×10^5^ cells/ml reservoir concentration, the values for homogeneous droplet occurrences were 4.7±0.6%, 9.0±1.9%, 12.7±1.6%, and 21.6±5.2% for 10%, 20%, 30%, and 50% target cell concentrations, respectively (**Fig. 3f**). Our results demonstrate that higher single cell encapsulation probability yields higher homogeneity as dictated by Poisson distribution and shown by experimental results.

**Figure 4:**
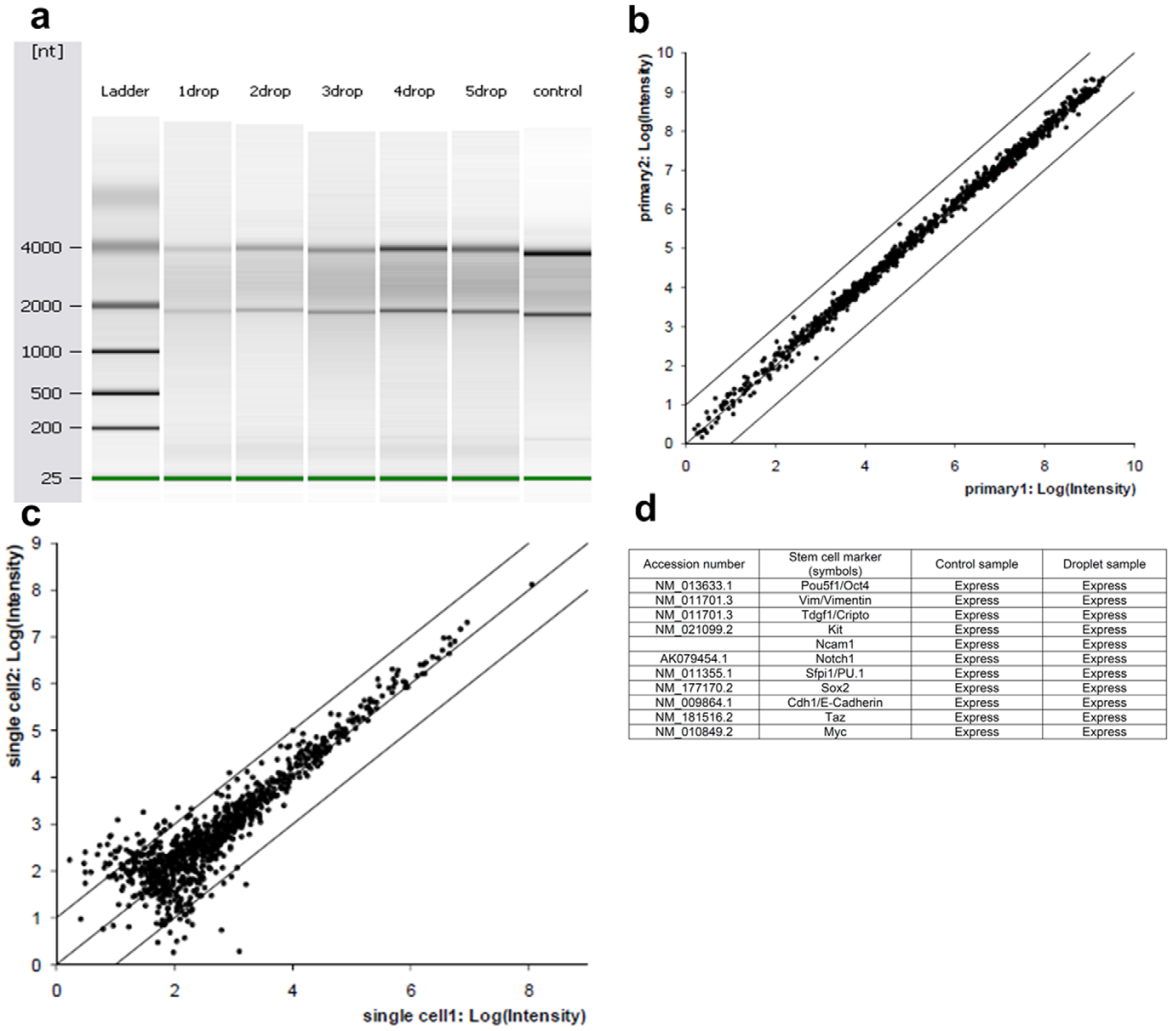
Total RNA expressions in printed droplets (virtual gel). (**a**) Total RNA quality on virtual gels (Agilent bioanalyser) as number of droplets increase. The 96-well plate was then placed underneath the ejector and one droplet was ejected into each of the first nine wells of the first row of wells. This procedure was repeated with additional four rows of the plate, where two, three, four and five droplets were ejected in each well resulting to a total of five groups of droplet samples, each group having an increasing droplet volume and increasing number of cells ranging from ten to fifty cells. For extraction purposes and consistency, before the RNA analysis was performed, each row was divided into three triplicates. Hence, the analysis was based on average 30, 60, 90, 120, and 150 cells for each triplicate for bioanalysis. To investigate nonprinted cells, 0.5 µl samples were drawn from the cell solution and pipetted into the three wells in each of the first three rows of the plate as controls. The controls had 1000 cells on average. The last lane showed the results for the control RNA which was prepared by a manual dilution method without ejection. (**b**, **c**) Comparison of the reproducibility of the RNA expression levels for (**b**) two replicates of the control set by the manual pipette method and (**c**) two experimental RNA samples obtained by droplet method. The scatter-plots were compared the reproducibility of expression level measurements for the 1000 genes with the highest expression levels in the experimental samples. (**d**) Expression of stem cell-related markers was examined to assess whether RNA obtained from the cell encapsulating droplets provided useful biological information in comparison to control samples. Our results showed that these 11 stem cell markers including Kit and Notch1, were found in both the patterned cells and the control groups.

Finally, we evaluated the sensitivity and reproducibility of a functional genomic analysis of stem cell encapsulating droplets. For these evaluations, we used DNA microarrays to measure gene expression levels in RNA samples extracted from mouse embryonic stem cells obtained by using drop-on-demand. We compared the results with RNA extracted from a control pool of mESCs, which were isolated with serial dilution and manual pipetting. The total RNA quality from the droplet-based isolated cells was similar to that of the control when assessed with the Agilent bioanalyser using the 28s/18s ratio (**Fig. 4a**). This demonstrated that RNA remains intact throughout the patterning process. In a genome-wide analysis, the 1000 genes with the highest expression levels were measured on DNA microarrays for the printed and control cells. The reproducibility of the gene expression is illustrated by the scatter plots of the microarray measurements on replicate samples (**Fig. 4b, c**). Although the expression levels exhibited greater variability at the lower expression levels than the control samples, the median coefficient of variation (CV) across three replicates was 5% in both cases. Stem cell-related markers were utilized to assess whether the RNA obtained from the printed cell group provided useful biological information in comparison to the control samples. Altogether eleven stem cell markers including Kit and Notch1 were found in both the printed cells and non-printed control groups (**Fig. 4d**). These results indicate that we were able to successfully isolate and analyze mRNA from cell encapsulating droplets for functional genomic studies. (See **Materials and Methods**, *Total RNA extraction and analysis*).

There are also limitations on the drop-on-demand approach for single cell sorting compared to the microfluidic deterministic cell encapsulation approaches. As the heterogeneity of sample increases, the types of cells that need to be tracked in the sample also increase for microfluidic systems. The outcome is that the tracking system requires more complex peripheral setups and it can require high-end computerized controls which has an impact on scalability [29], [30], [31]. The drop-on-demand cell encapsulation approach presented here has a trade-off from a deterministic aspect, but on the other hand it offers lesser handling steps to sort and pattern cells, where cells might be affected by the conditions in the physical environment. For single cell encapsulation, microfluidic method is more deterministic and may provide a better control over cell encapsulation. Further, the deterministic approach offers an efficient system to handle minimum number of cells, i.e. single to ten cells. Second, microfluidic approach can be convenient in case an integrated on-chip experiment requires further cell handling steps after sorting such as on-chip polymerase chain reactions (PCR). Finally, throughput of drop-on-demand sorting process is limited by the parallel printing setup and the wide field of view imaging method.

We demonstrated a high-throughput drop-on-demand single cell isolation technology and presented how it fits to the statistical models. Our data revealed that the drop-on-demand single cell patterning platform can isolate viable target stem cells from a heterogeneous sample. In addition, this patterning approach can be adapted to generate multiple droplet arrays in parallel, further enhancing throughput. This work marks the first genomic analysis study on printed cells. Furthermore, we showed that functional genomic information, specifically mRNA expression levels, obtained from ejected cells was preserved throughout the entire process.

## Materials and Methods

### Cell preparation and staining

The work space is a HEPA filter equipped sterile hood (Cleanroom International, 13202). All materials were decontaminated with 70% ethanol and RNaseZAP® (Applied Biosystems) prior to introducing the cell solutions. Mouse embryonic stem cells (mESCs, wild type R1 cell line, ∼20% confluent) [38] were trypsinized (10×0.5 Trypsin-EDTA, Gibco, 15400) and passaged from Corning® flask (Corning, CLS3150) into a Falcon™ tube (BD, 352096). The cell solution was centrifuged at 1000 rpm for 3 minutes and cells were washed with DPBS (Dulbecco's phosphate buffered saline, Dulbecco, P1010) and resuspended in culture medium. The culture medium was prepared from 500 ml Knockout ES Media (Gibco, 11965-092), 50 ml ES FBS (Gibco, 10439-024), 1 ml 2-Mercaptoethanol (Sigma, P4333), 5 ml NEAA (Non-essential amino acid, Sigma, M7145), 5 ml L-Glutamin (Gibco, 25030), 2 µl LIF/ml (Leukemia inhibitory factor, Millipore, ESGRO®), and 5 ml Pen/Strep (Sigma, P4333). All components were passed through a sterile filter (500 ml Express Plus 0.22 µm membrane, Millipore, SCGPU05RE). A sample/aliquot of the cell solution was stained with 0.4% Trypan Blue solution (Invitrogen, 15250061) and counted with a hemacytometer (Hausser Scientific, 1483). Two sets of low (1×10^5^, 1.5×10^5^, and 2×10^5^ cells/ml) and high (2.5×10^5^, 5.0×10^5^, and 7.5×10^5^ cells/ml) cell concentrations were prepared. Using a molecular probes Live/Dead assay (Invitrogen, L-2334) for mammalian cells, pre- and post-ejection cell viabilities were recorded. Also, to make a heterogeneous cell mixture that contains both stained and non-stained cells, two samples of each concentration of cells were prepared and stored in separate test tubes. After staining with a Live/Dead assay and washing with DPBS to remove excessive staining solution, the stained and unstained cells were mixed to prepare 10% to 50% volume fraction heterogeneous solutions. These solutions served as models that represent different fractions of target cells. Pre-ejection cell viability was measured from samples taken directly from the cell solutions (see **Fig. S4** online). Post-ejection cell viability was measured from droplets ejected through the 150 µm valve orifice with a frequency ranging from 1 Hz to 1 kHz. The pulse duration was 55 µs to 65 µs, at 34.5 kPa of gas pressure.

### Drop-on-demand single cell patterning

Drop-on-demand can generate droplets both with spatial control over position and temporal control over ejection. The single cell droplet ejector system consists of an automated xyz stage (NLS4 Series Precision Linear Stage, Newmark systems Inc.) and a sub-nanoliter dispensing valve (TechElan LLC, G100), which were synchronized with a control program, Labview™ (Labview, National Instruments Corporation) (see **Fig. S1a** online). The spatial resolution and repeatability of the stage are 0.13 µm and 5 µm, respectively. To image *in-situ* cell encapsulating droplets during the ejection process, a CCD camera (Edmund optics, EO-1312M) equipped with a 0.5× lens (INFINITUBE FM-200 (NT58-309, 2×) and a PL/FD-195 OBJ objective (NT59-115, 0.25×, Edmund optics) was combined with a synchronized light controller (S4000 strobe controller, Edmund optics) for stroboscopic illumination. The overall system, i.e. xyz stage, sub-nanoliter dispensing valve, and stroboscopic light controller, was synchronized and programmed by a PC-based DAQ control board (NI cDAQ-9172 and NI-9401, National Instruments). Pressure regulated nitrogen gas was connected to a syringe reservoir through an adapter cap (KDS503S6, Techni-Tool Inc.), and the syringe was connected to the sub nanoliter dispensing valve by tubing (Tygon® tube, Fisher scientific Inc.).

A heterogeneous sample comprising a mixture of cells and cell media solution was loaded into a 1 ml syringe reservoir and each droplet was printed at pre-determined positions on a glass surface or into a 96-well plate. The number of cells per droplet was primarily determined by the droplet size and the cell concentration in the printing solution. Prior to taking a 100 µL cell solution, the Falcon tube was vortexed. The reservoir was stirred also after loading samples to maintain an even cell distribution within the medium. High-frequency actuation (1 kHz) was used to eject the excess cell solution, after which the valve was washed out with DI water (∼60°C). We calibrated droplet size and maintained ejection stability to ensure that the orifice was free of cell debris and medium residues. Once this process was completed, 70% ethanol was ejected through the valve, leaving a small amount of ethanol in the syringe and valve until the next experiment. This ensured clean, repeatable, and reliable operation. The size of each droplet was determined by stroboscopic images during stable droplet formation. Adjustable ejection parameters were xyz stage speed, valve opening frequency and duration, and gas pressure. The valve opening time and gas pressure were regulated to control the droplet size and number of cells per droplet. A droplet size measurement was conducted both in air and on the glass substrate to provide feedback to the manual/automatic droplet size controller. Droplet stability was tested using 90 µs valve opening time and 34.5 kPa pressurized nitrogen gas. Stroboscopic images of ejected droplets were taken every second (see **Fig. S2a** online). If the ejector was not operated in a stable regime, satellite droplets were generated during ejection. Control over stable droplets without satellites was maintained at optimized ejection conditions, *i.e.* 64 µs valve opening time, 34.4 kPa pressurized nitrogen gas, and 25 Hz ejection frequency (see **Fig. S2b** online). The size of the ejected droplets was investigated in air and at the receiving surface of a slide glass substrate by ejecting 100 droplets. Three pressure and ejection conditions were used at 34.4 kPa of air pressure for pulse durations of 55 µs, 60 µs and 65 µs, yielding droplets of different sizes. As shown in **Figure 2b**, we ejected multiple droplets under same ejection conditions to investigate the droplet size uniformity. A liquid nitrogen bath was prepared by inserting a 60 mm (D)×15 mm (H) Petri dish inside a Styrofoam box, filling the dish and surrounding box area with liquid nitrogen, and placing the box 20 mm underneath the valve. A fluorescent microscope (Eclipse TE-2000 U, Nikon) equipped with a CCD camera (Spot RT-KE Mono, RT700, Diagnostic Instruments, Inc.) was paired with a software (Spot Advanced, Diagnostic Instruments, Inc.) to capture images of the frozen droplets at 10× and 20× magnification. Droplet diameters were obtained with the software by fitting circles around each droplet image (Spot Advanced). To measure the diameter of the ejected droplets on the surface (see **Fig. S2c** online), the same stroboscopic image setup was used after changing the lens to a 0.25× magnification tube (INFINITUBE FM-200 (NT58-309, 2×) and a PL/FD-390 OBJ objective (NT59-117, 0.125×), Edmund optics).

Droplet arrays (50×50 in size) were patterned at 25 Hz at 34.5 kPa of nitrogen gas pressure onto a 75 mm×50 mm glass microscope slide. The distance between the valve ejector and the slide was 1.5 mm. The drop-to-drop distance was determined to be larger than the droplet size to avoid overlapping patterns. The microscope slide was then placed inside a covered 100 mm (diameter)×15 mm (height) Petri dish. To avoid droplet evaporation while imaging, PBS surrounding the patterned droplet area was used to create a local humid environment. The droplet array was searched for target cells using a fluorescent microscope with an automatic stage (Axio observer equipped with Axio-Cam MRm, Carl Zeiss Micro-Imaging GmbH). Standard deviations were calculated from the average number of cells encapsulated per droplet on the patterned droplet array (see **Fig. S3** online).

### Total RNA extraction and analysis

To prevent contamination RNase AWAY® (Sigma Aldrich), a ribonuclease decontaminant, was ejected through the valve, followed by nuclease-free water (Applied Biosystems) in preparation for cell encapsulating droplet ejection. Then, the entire area surrounding the ejector system was sprayed and wiped with RNaseZAP®. Three rows in a 96-well plate were filled with 100 µL of RLT Buffer solution (QIAGEN, 1% mercaptoethanol) to lyse the ejected cells and to preserve the RNA. The 96-well plate was then placed underneath the ejector and one droplet was ejected into each of the first nine wells of the first row of wells. This procedure was repeated with additional four rows of the plate, where two, three, four and five droplets were ejected in each well resulting to a total of five groups of droplet samples, each group having an increasing droplet volume and increasing number of cells ranging from ten to fifty cells. For consistency of bioanalysis, before the RNA analysis each row was divided into three triplicates. Hence, the analysis was based on average 30, 60, 90, 120, and 150 cells for each triplicate for bioanalysis. To investigate nonprinted cells, 0.5 µl samples were drawn from the cell solution and pipetted into the three wells in each of the first three rows of the plate as controls. The controls had 1000 cells on average. Qiagen RNeasy Minikit (QIAGEN) was used to purify the total RNA. From the 96 well plate sample, triplicates were used to form single 300 µl samples, after which each sample was transferred into a QIAshredder column in a 2 ml collection tube. The lysate was homogenized by placing the tubes in a microcentrifuge for 2 minutes at 5000 rpm. Each flow-through was transferred to a fresh 1.5 ml eppendorf tube and 300 µl of 70% EtOH was added to each tube and mixed by pipetting. Each lysate solution was then applied to an RNeasy mini column in a 2 ml collection tube and spun for 15 seconds at 10,000×g, discarding the flow-through afterwards. A buffer, 350 µL, RW1 (QIAGEN, RNeasy Minikit) was added to each column, and the columns were centrifuged for 15 seconds at 10,000×g, discarding the flow-through afterwards. This step was repeated. Then the columns were transferred to new 2 ml catch tubes. Next, 500 µL of buffer RPE (QIAGEN, RNeasy Minikit) was pipetted into each RNeasy column, and the tubes were centrifuged for 15 seconds at 10,000×g (gravity), decanting the flow-through afterwards. Again, 500 µL of RPE buffer was pipetted into the tubes, and the columns were centrifuged for 15 seconds at 10,000×g and placed in new 2 ml catch tubes and spun at full speed for one minute to ensure that the membranes were dry. The columns were then transferred to new 1.5 ml collection tubes. The tubes were left at room temperature for one minute after pipetting 52 µL of ribonuclease-free water directly onto each membrane and then centrifuged for 1 minute at 10,000×g. The elution was repeated with the same volumes into the same tubes. The resulting eluant was speed vacuumed for 45 minutes at room temperature, and all samples were equilibrated to 15 µL. With the 2100 Expert® software, the isolated RNA was analyzed for quantity and quality using the Agilent 2100 Bioanalyzer (Agilent Technologies, Palo Alto, CA, using the RNA 6000 Pico Series II assay). All data is MIAME compliant and that the raw data has been deposited in a MIAME compliant database (GEO accession ID: GSE24330).

The total RNA was amplified with Ambion's Illumina TotalPrep RNA amplification kit (Ambion, Inc. Austin, TX). A brief description of the amplification/purification process is as follows: Reverse transcription to synthesize first strand cDNA. 2–3 ng sample (total RNA) is primed with the T7 oligo (dT) primer to synthesize cDNA containing a T7 promoter sequence. Second strand cDNA synthesis converts the single-stranded cDNA into a double-stranded DNA (dsDNA) template for transcription. The reaction employs DNA polymerase and RNase H to simultaneously degrade the RNA and synthesize second strand cDNA. Next, cDNA purification removes RNA, primers, enzymes, and salts that would inhibit *in vitro* transcription. *In vitro* transcription to synthesize cRNA generates multiple copies of biotinylated cRNA from the double-stranded cDNA templates. This is the amplification and labeling step. Then cRNA purification removes unincorporated NTPs, salts, enzymes, and inorganic phosphate. After purification and fragmentation, the cRNA is ready to use with direct hybridization array kits. For hybridization to arrays (AppliedMicroarrays CodeLink Mouse Whole Genome Bioarray), 10 µg of labeled cRNA was fragmented, denatured at 90°C for 5 min, and then hybridized at 37°C for 20 hours. The arrays were then washed in buffer at 47°C for 60 minutes. After washing, the arrays were incubated at room temperature for 30 minutes with a fluorophore (Streptavidin-Alexa Fluor 647, Invitrogen Eugene, OR), run through a series of 5 minute rinses, spun dry, then scanned on a GenePix 4000B scanner (Axon Instrument, Sunnyvale, CA). The Mouse Whole Genome Bioarrays contains 38,313 single 30-mer oligonucleotide probes. For the gene expression measurements were normalized to a median expression level of 1.0 across the array. 1000 genes with the highest expression levels “>1” in the experimental samples was used for comparisons. Three Bioarrays were run for both the experimental and control samples to enable statistical analysis of the reproducibility of the gene expression measurements for the most abundant genes.

### Statistical modeling

Statistics is widely adapted to biological analysis. Number of cells used in experiments are large, and these experiments follow the law of large numbers (LLN) and sampling processes are typically random, i.e. simple random sampling (SRS) [33], [34], [35]. In our statistical modeling, we described cell encapsulation as a random process with three random variables and their probability distribution functions (PDFs) where cells are encapsulated in droplets by a mechanical valve from a heterogeneous cell mixture. We followed three steps to model our target single cell encapsulation process. Based on experimental results, we (i) defined our system as a random system with random variables, (ii) checked whether each process was biased or un-biased, (iii) established statistical models with respect to each process and found parameters for the statistical models, e.g. mean (μ), variation (σ), and Poisson distribution parameter (λ), and (iv) evaluated overall system efficiency based on both CLT and SRS. Following these steps, we modeled our system as a random process (see **Table S1 and Fig. S5** online).

## Supporting Information

Figure S1 **Homogeneous single cell droplet sorting system.** A computerized xyz stage was synchronized with a pulse controller, Labview™. The automated stage positioned the substrate with 5 µm spatial resolution. Two ejectors and a 10× camera permitted *in-situ* imaging and lysing of the droplets. Schematic of droplet ejector showed cells flowing into the valve driven by a controlled air pressure pulse (34 kPa for 55 µs). A heterogeneous sample, mixture of cells and media solution, was loaded into a reservoir. Each droplet was placed at a predetermined position.
(TIF)

Figure S2 **Controlling droplet size.** Image and illustration of ejected droplet in air were shown. (**a**) Stroboscopic images of ejected droplets. When the valve opening time and the gas pressure is not optimized, we observed satellite droplets. The sequential images were taken every second. (**b**) Stable droplets, D_air_ = 245±12 µm (V≈7.7±1.2 nl), were generated during optimized ejection: *i.e.* 55 µs valve opening time, 34.4 kPa pressurized nitrogen gas, and 25 Hz ejection frequency. The droplet size was measured at 2.3 mm distance from the ejector. (**c**) Droplet size at bottom surface. After landing on the surface, the diameter of the ejected droplet was determined by the surface tension and droplet volume. H_droplet_ = 68±2 µm, D_droplet_ = 525±15 µm, (V≈7.6±0.6 nl). Scale bars are 1 mm in **a** and **b**, 100 µm in c, respectively.
(TIF)

Figure S3 **High throughput droplet patterning system, images of printed droplet array.** One hundred droplets were inspected for target homogeneous droplet sorting. (**a–d**) droplet array was generated under four different conditions, (**a**) 1×10^5^ cells/ml with 10% target cell concentration, (**b**) 1×10^5^ cells/ml with 50% target cell concentration, (**c**) 2×10^5^ cells/ml with 10% target cell concentration, (**d**) 2×10^5^ cells/ml with 50% target cell concentration. Scale bar, 1 mm.
(TIF)

Figure S4 **Pre- and post-patterning cell viability.** (**a**) Normalized cell viability was obtained comparing the two viabilities. Average and standard deviation of normalized viability were 96.4±0.8%, 97.9±2.3%, 97.2±2.2%, and 96.0±2.4% from 0.5×10^5^ to 2×10^5^ cells/ml of cell loading concentrations (n = 3 sets left y-axis reference). (**b**) Pre-patterning (flask) viabilities were measured based on 200 cells in a 10 µl sample volume. Post-print cell viabilities were obtained from 2500 droplets for each cell loading concentration, *i.e.* 70 cells at 0.5×10^5^ cells/ml and 575 cells at 2×10^5^ cells/ml. Overall mean and standard deviation of pre- and post-printing cell viabilities were 96.7±0.5% and 93.8±1.1%, respectively (right hand y-axis).
(TIF)

Figure S5 **Probability of encapsulating a single target cell in a droplet was presented by probability distribution functions, P(** ***X*** **=** ***X*** **_single target cell_).** (**a**) The number of homogeneous droplets was modeled using Poisson distribution in a random variable space, *i.e.* number of target cells. The model was verified using a coefficient, λ, and experimental results. (**b**) According to four different cell loading concentrations and target cell concentrations, 16 Poisson distributions resulting from 4 cases of concentration times 4 cases of target cell mixture concentrations and their coefficients, λ, were obtained comparing to experimental results. As cell loading density increases, target cell concentration, λ values increase, λ_max_ = 0.95 and λ_min_ = 0.03. Based on the analysis, statistical models for 1.0×105 cells/ml with 10% target cell mixture were based on λ = 0.05.
(TIF)

Table S1 **Statistical modeling results for drop-on-demand target single cell encapsulation.** (**a**) Randomness of process was verified by three variables, number of samples (n), tolerance (ε), and confidence level (1−α) using an inequality^(**)^. Following the law of large numbers (LLN), minimum sample number was decided as 100 droplets for 90% confidence level and 15% tolerance. This sampling volume of a droplet represented (0.76 µl = 10×10×7.6 nl) 0.76% of the total volume of the ejection reservoir (0.1 mL). (**b**) Random processes have different probability distribution functions in accordance with their parameters, *i.e.* number of cell containing droplets (*X_drop_*), number of cells in a droplet (*X_cell_*), number of target cells per droplet (*X_target cell_*), and number of single target cell containing droplets (*X_single target cell drop_*). These four different random variables are represented by three PDFs to statistically model the cell encapsulation process, i.e. binomial, Poisson, and normal distributions. We investigated probability values and parameters, λ, for each case with respect to the cell loading concentrations, cell volume fraction, and percentage of target cells. (**c**) In the case of simple random sampling (SRS) process, statistical characteristics of a small sampling volume could represent the characteristics of a large population based on the central limit theorem (CLT). In our experiments, the target cell fraction (*F*_%_) shows same concentration as the reservoir concentration for 10% to 50% at 1.0×10^5^ cells/ml concentration (C_opt_) under conditions of 90% confidence level and 15% tolerance.
(DOCX)
