# Supplementary figures and images for: Drop-on-Demand Single Cell Isolation and Total RNA Analysis
Source: PLoS One. 2011 Mar 11;6(3):e17455. doi: 10.1371/journal.pone.0017455 (PMC3055874; doi:10.1371/journal.pone.0017455)

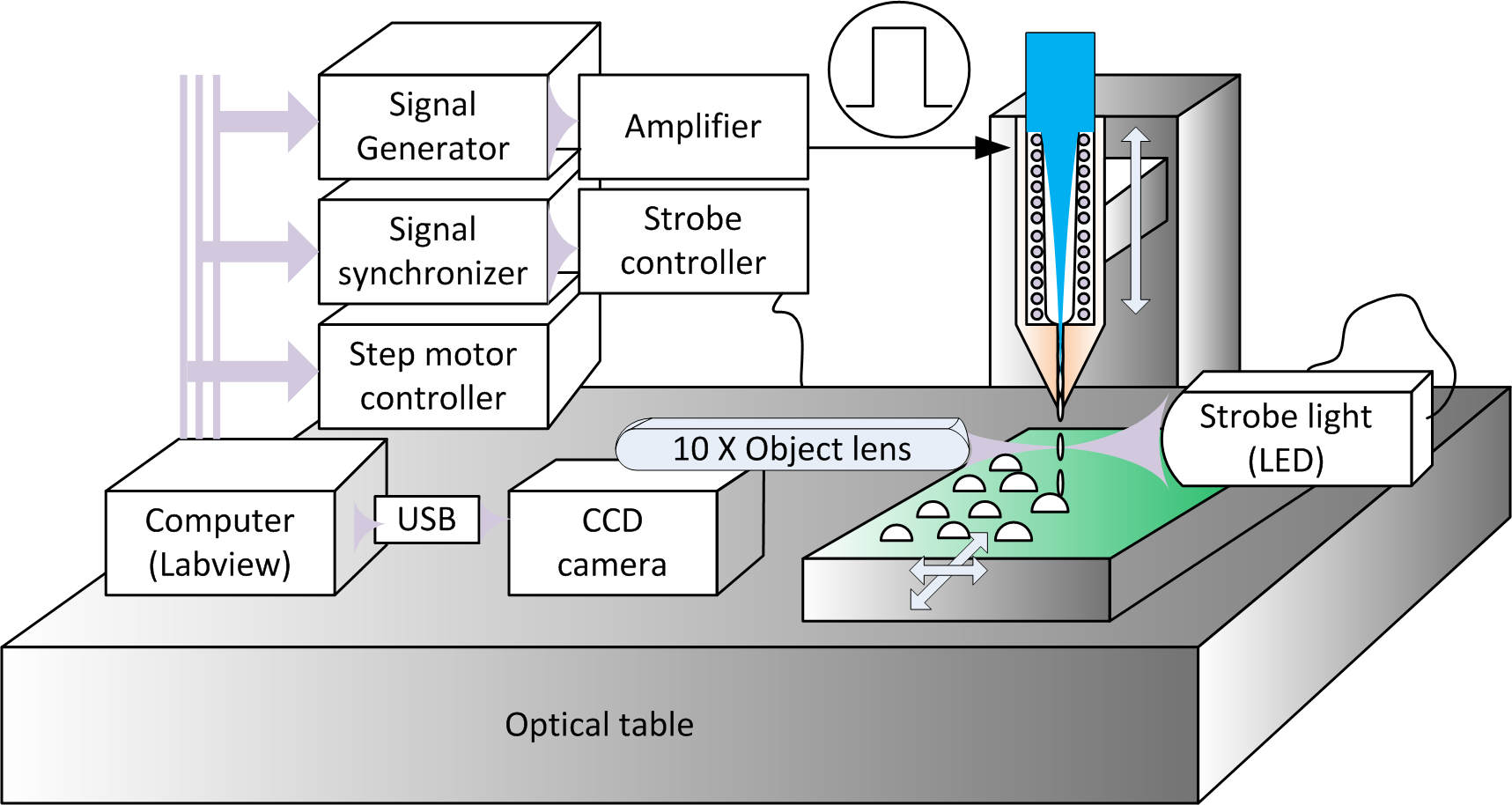

Supplement: Figure S1 — Homogeneous single cell droplet sorting system. A computerized xyz stage was synchronized with a pulse controller, Labview™. The automated stage positioned the substrate with 5 µm spatial resolution. Two ejectors and a 10× camera permitted in-situ imaging and lysing of the droplets. Schematic of droplet ejector showed cells flowing into the valve driven by a controlled air pressure pulse (34 kPa for 55 µs). A heterogeneous sample, mixture of cells and media solution, was loaded into a reservoir. Each droplet was placed at a predetermined position. (TIF) [file pone.0017455.s001.tif]

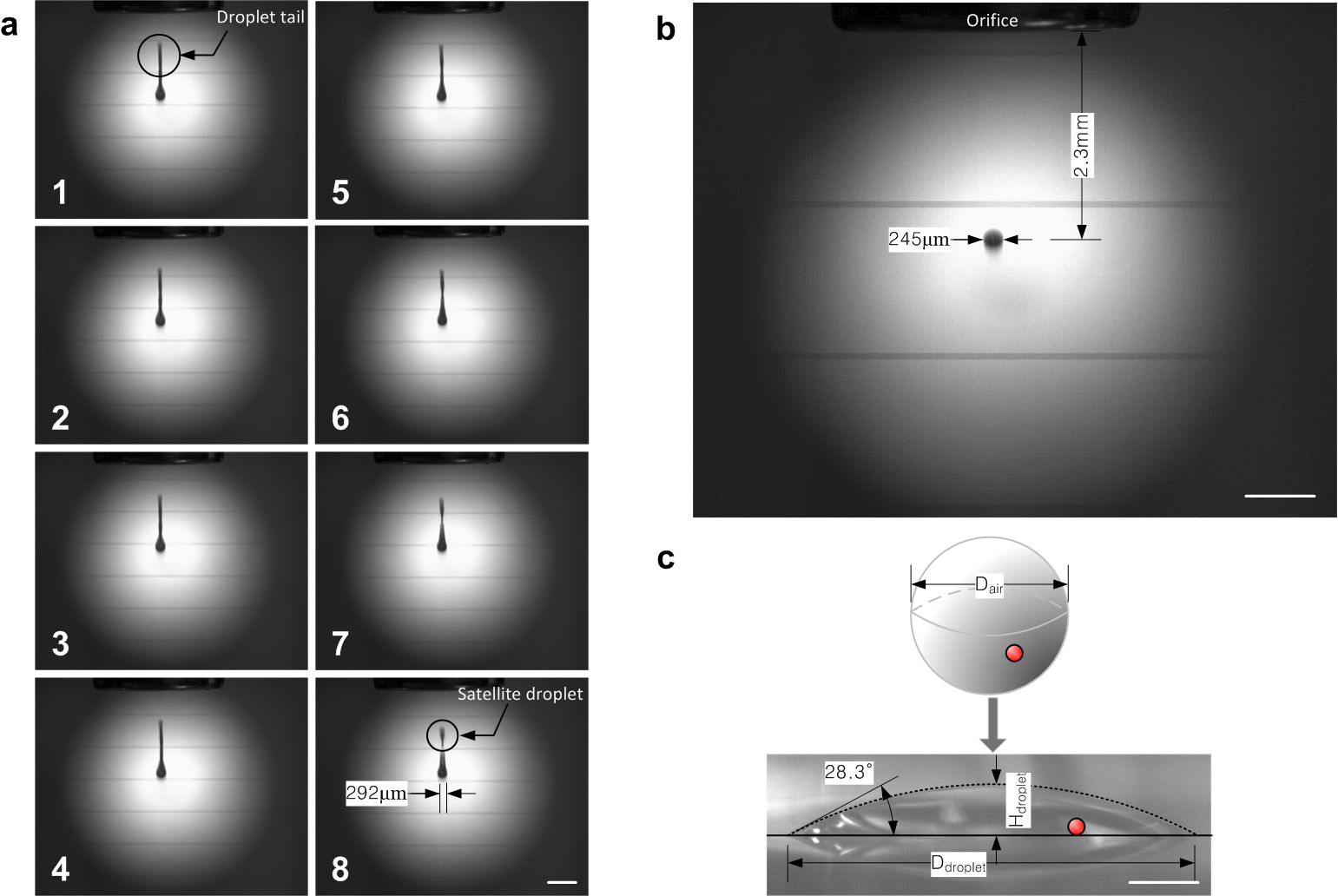

Supplement: Figure S2 — Controlling droplet size. Image and illustration of ejected droplet in air were shown. (a) Stroboscopic images of ejected droplets. When the valve opening time and the gas pressure is not optimized, we observed satellite droplets. The sequential images were taken every second. (b) Stable droplets, Dair = 245±12 µm (V≈7.7±1.2 nl), were generated during optimized ejection: i.e. 55 µs valve opening time, 34.4 kPa pressurized nitrogen gas, and 25 Hz ejection frequency. The droplet size was measured at 2.3 mm distance from the ejector. (c) Droplet size at bottom surface. After landing on the surface, the diameter of the ejected droplet was determined by the surface tension and droplet volume. Hdroplet = 68±2 µm, Ddroplet = 525±15 µm, (V≈7.6±0.6 nl). Scale bars are 1 mm in a and b, 100 µm in c, respectively. (TIF) [file pone.0017455.s002.tif]

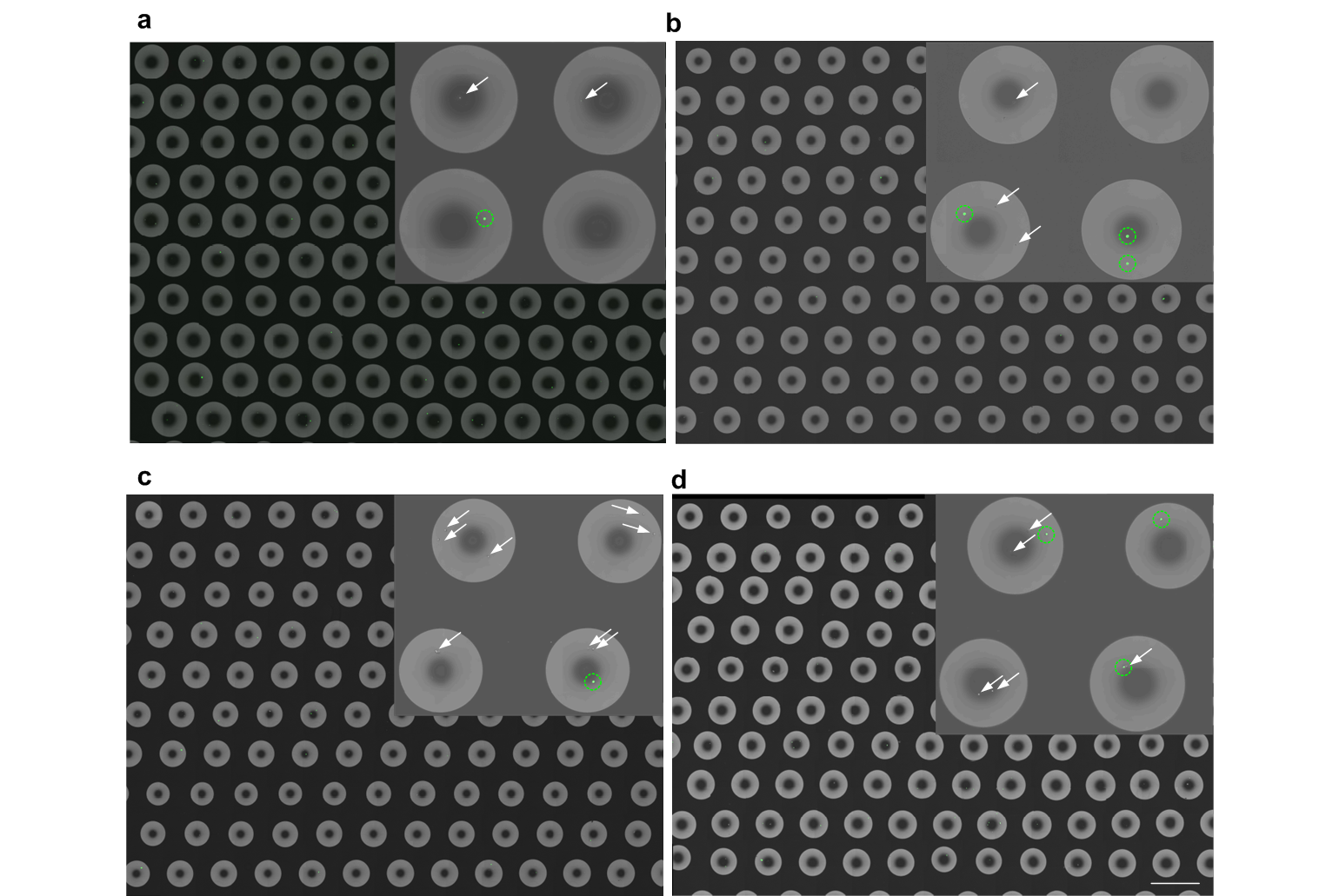

Supplement: Figure S3 — High throughput droplet patterning system, images of printed droplet array. One hundred droplets were inspected for target homogeneous droplet sorting. (a–d) droplet array was generated under four different conditions, (a) 1×10^5^ cells/ml with 10% target cell concentration, (b) 1×10^5^ cells/ml with 50% target cell concentration, (c) 2×10^5^ cells/ml with 10% target cell concentration, (d) 2×10^5^ cells/ml with 50% target cell concentration. Scale bar, 1 mm. (TIF) [file pone.0017455.s003.tif]

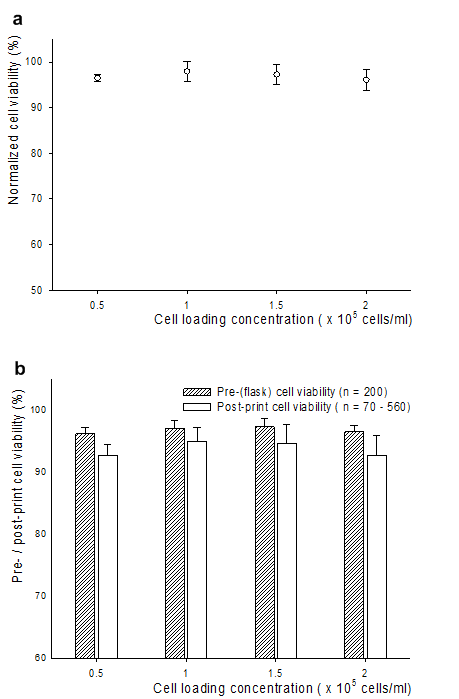

Supplement: Figure S4 — Pre- and post-patterning cell viability. (a) Normalized cell viability was obtained comparing the two viabilities. Average and standard deviation of normalized viability were 96.4±0.8%, 97.9±2.3%, 97.2±2.2%, and 96.0±2.4% from 0.5×10^5^ to 2×10^5^ cells/ml of cell loading concentrations (n = 3 sets left y-axis reference). (b) Pre-patterning (flask) viabilities were measured based on 200 cells in a 10 µl sample volume. Post-print cell viabilities were obtained from 2500 droplets for each cell loading concentration, i.e. 70 cells at 0.5×10^5^ cells/ml and 575 cells at 2×10^5^ cells/ml. Overall mean and standard deviation of pre- and post-printing cell viabilities were 96.7±0.5% and 93.8±1.1%, respectively (right hand y-axis). (TIF) [file pone.0017455.s004.tif]

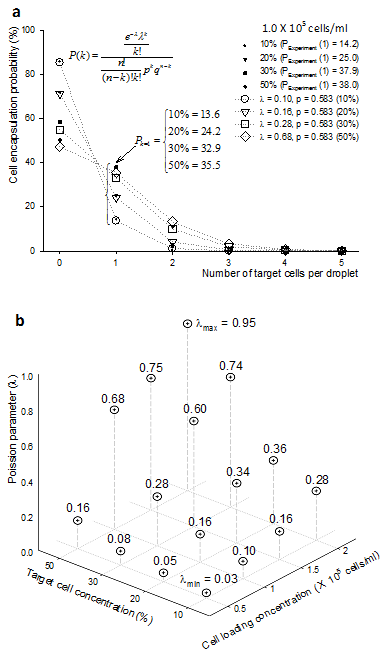

Supplement: Figure S5 — Probability of encapsulating a single target cell in a droplet was presented by probability distribution functions, P( X = X single target cell). (a) The number of homogeneous droplets was modeled using Poisson distribution in a random variable space, i.e. number of target cells. The model was verified using a coefficient, λ, and experimental results. (b) According to four different cell loading concentrations and target cell concentrations, 16 Poisson distributions resulting from 4 cases of concentration times 4 cases of target cell mixture concentrations and their coefficients, λ, were obtained comparing to experimental results. As cell loading density increases, target cell concentration, λ values increase, λmax = 0.95 and λmin = 0.03. Based on the analysis, statistical models for 1.0×10^5^ cells/ml with 10% target cell mixture were based on λ = 0.05. (TIF) [file pone.0017455.s005.tif]
