# Supplementary material for: Drop-on-Demand Single Cell Isolation and Total RNA Analysis
Source: PLoS One. 2011 Mar 11;6(3):e17455. doi: 10.1371/journal.pone.0017455 (PMC3055874; doi:10.1371/journal.pone.0017455)
Supplement: Table S1 — Statistical modeling results for drop-on-demand target single cell encapsulation. (a) Randomness of process was verified by three variables, number of samples (n), tolerance (ε), and confidence level (1−α) using an inequality(**). Following the law of large numbers (LLN), minimum sample number was decided as 100 droplets for 90% confidence level and 15% tolerance. This sampling volume of a droplet represented (0.76 µl = 10×10×7.6 nl) 0.76% of the total volume of the ejection reservoir (0.1 mL). (b) Random processes have different probability distribution functions in accordance with their parameters, i.e. number of cell containing droplets (Xdrop), number of cells in a droplet (Xcell), number of target cells per droplet (Xtarget cell), and number of single target cell containing droplets (Xsingle target cell drop). These four different random variables are represented by three PDFs to statistically model the cell encapsulation process, i.e. binomial, Poisson, and normal distributions. We investigated probability values and parameters, λ, for each case with respect to the cell loading concentrations, cell volume fraction, and percentage of target cells. (c) In the case of simple random sampling (SRS) process, statistical characteristics of a small sampling volume could represent the characteristics of a large population based on the central limit theorem (CLT). In our experiments, the target cell fraction (F %) shows same concentration as the reservoir concentration for 10% to 50% at 1.0×10^5^ cells/ml concentration (Copt) under conditions of 90% confidence level and 15% tolerance. (DOCX) [file pone.0017455.s006.docx]

|  | **Process description** | **Variable** | **PDF^(1)^** | **Results** |
| --- | --- | --- | --- | --- |
| (a) | Randomness (cell encapsulation) | *n,* *ε, α* | Based on normal distribution (LLN) | - number of samples: 10 × 10 |
|  |  |  |  | - sampling volume fraction^(2)^: 0.76% |
|  |  |  |  | - confidence level: 90% |
|  |  |  |  | - tolerance: 15% |
| (b) | Number of cell containing droplets (cell encapsulation) | *X_drop_* | Binomial distribution | *- ^(*)^ f(P)* = 27 ~ 87% |
|  |  |  |  | - cell loading concentration |
|  |  |  |  | - cell volume fraction^(3)^ |
|  | Number of cells in a droplet (cell distribution) | *X_cell_* | Binomial distribution, Poisson distribution | - *f(λ)* = 0.4 ~ 2.2 |
|  |  |  |  | - cell loading concentration |
|  | Number of target cells in a droplet (target cell distribution) | *X_target cell_* | Poisson distribution | *- f(λ)* = 0.03 ~ 0.95 |
|  |  |  |  | - cell loading concentration |
|  |  |  |  | - percentage of target cells |
|  | Number of single target cell containing droplets (target droplet distribution) | *X_single target cell drop_* | Normal distribution | *- f(λ)* = 0.03 ~ 0.95, P = 27 ~ 87% |
|  |  |  |  | - cell concentration |
|  |  |  |  | - percentage of target cells |
| (c) | CLT for SRS^(4)^ (target droplet selection) | *ε, α, C_opt_, F_%_* | Based on normal distribution (CLT for SRS) | - confidence level: 90% |
|  |  |  |  | - tolerance: 15% |
|  |  |  |  | - optimum cell concentration: 1.0 × 10^5^ |
|  |  |  |  | - target cell fraction^(5)^: 10 ~ 50% |

^(*)^*f(x)*: functions for variable *X*

^(**)^ Sampling number: n ≥ pq/ε^2^α, where, p = 0.6, q = 0.4 (ref. 41)

^(1)^PDF: Probability Distribution Function

^(2)^Sampling volume fraction (%) = total sample set volume / entire volume (100 µl) × 100 = 0.76%

^(3)^Cell volume fraction = number of cells × cell volume / droplet volume

^(4)^CLT (Central Limit Theorem) and SRS (Simple Random Sampling)

^(5)^Target cell fraction = number of target cells / number of whole cells
